# Resource Prospects of Municipal Solid Wastes Generatedin the Ga East Municipal Assembly of Ghana

**DOI:** 10.5696/2156-9614-7.14.37

**Published:** 2017-06-22

**Authors:** Benedicta Abiti, Susanne Hartard, Heike B. Bradl, Davar Pishva, John Kojo Ahiakpa

**Affiliations:** 1 Department of Family and Consumer Sciences, College of Basic and Applied Sciences, University of Ghana, Legon-Accra, Ghana; 2 Institute for Applied Material Flow Management, Trier University of Applied Sciences, Umwelt Campus, Birkenfeld, Germany; 3 Graduate School of Asia Pacific Studies, Ritsumeikan Asia Pacific University (APU), Beppu, Japan; 4 Research Desk Consulting Ltd., Kwabenya-Accra, Ghana

**Keywords:** waste flow analysis, sustainable waste management, municipal solid waste, circular economy

## Abstract

**Background.:**

Municipal solid wastes management has recently become an important public health concern. Municipal solid wastes are a major source of raw materials that could be used for resource recovery for diverse applications.

**Objectives.:**

The present study aimed to determine the composition of municipal solid waste and recoverable resources from the waste of the Ga East Municipal Assembly (GEMA) in the Greater Accra region of Ghana.

**Methods.:**

An exploratory approach was used to collect pertinent data from the Abloradgei dumpsite in GEMA using semi-structured interviews and focus group discussion. A field characterization study was undertaken to segregate and estimate the value of various components of collected waste. Dumpsite workers were asked about current general composition of MSW, mode of collection and disposal, record of sanitation-related diseases, use of modern treatment plant, waste management legislation and enforcement challenges, number of trucks received by the dumpsite per day, record on pretreatment of MSW before disposal, and use of personnel protective equipment.

**Results.:**

The results showed that significant proportions (48.8%) of the municipal solid wastes were organic materials, while the remaining (51.2%) were inorganic materials. The results also showed that 63% of the municipal solid waste is collected with no sorting from the source and no modern treatment applied before dumping. It was estimated that the value of the recyclable materials in GEMA municipal solid waste amounts to Ghana Cedis (GH¢) 9,381,960 (plastic); 985,111 (mixed glass); 5,160,078 (paper) and 11,586,770 (metal) with a total of GH¢ 27,113,919 ($10,845,568) equivalent to 2,106,339.2 m^3^ (74,384,667.5 ft^3^) per annum of biogas from these components with a market value of GH¢ 1,997,972.17 ($768, 393.62); 11,579 Mwh (1.32 Mw) of electricity and 9,535 Mwh (1.09 Mw) of heat. This is estimated to be lost with the current waste management practices.

**Conclusions.:**

We recommend that GEMA institute sustainable recycling practices and utilization of biogas production technologies and prioritize sanitation and waste management education for the public, obligate home segregation of waste materials, involve workers by providing them with protective clothing, incorporate informal waste collectors and scavengers into the new system and collaborate with research institutions in waste-to-resource projects to ensure a more sustainable waste management system in the municipality.

**Participant Consent:**

Obtained

## Introduction

The ongoing rise in rural-urban migration has quadrupled the rate of urbanization of cities and metropolitan areas in developing countries. This has fueled the accumulation of waste across towns and cities, exacerbating the problem of solid waste management in urban Africa.^1^ The World Bank has warned that the amount of garbage generated globally is escalating so fast that they estimate that the amount of waste produced by this growing urban population will triple by 2050. It has been estimated that the 3.5 million tons of daily global waste produced will rise to six million tons per day by the year 2025.^2^ The World Health Organization stated that even though tremendous progress has been made, the world has failed to meet the United Nations Millennium Development Goal 7, Ensuring Environmental Sustainability's Target C on sanitation, tailored at halving the proportion of people without access to improved sanitation by 2015.^3^

The pursuit of development is also associated with a rise in the material living standards of people and changing trends in demand for goods and services, contributing to the proportionate increase in per capita waste generation and swelling levels of inappropriately disposed waste.^4^ Presently, several countries are grappling with a looming waste management crisis as existing landfills have either reached or are nearing their limits and posing environmental risks.^5^

The production of waste is an inevitable facet of human existence since waste is a consequence of the daily lifestyle of all humans.^6^ Nevertheless, increasing population and its associated problems can make the situation worse in the absence of sustainable waste management systems.^7^ Every sector of the national economy contributes to the mounting mass of waste.^8^ Municipal solid waste management is a major drain on governments' budgets (about 20–30% of municipal budgets) in developing countries.^9^ However, the Ga East Municipal Assembly (GEMA) collects less waste than is generated. Waste has become a major bottleneck for the Ghanaian economy and health institutions, as well as environmental agencies, compelling the president to declare the first Saturday of every month as a National Sanitation Day in 2014 to address the embarrassingly poor sanitation situation of the country.^10^ The country is currently overwhelmed with copious amounts of waste, particularly in major cities.^11^

Landfilling, composting and incineration are well-developed waste management techniques commonly employed for solid waste management. However, sophisticated and advanced incineration plants with no spillage of smoke has made garbage incineration an attractive and more environmentally friendly waste management technique.^12^ Waste incineration promotes resource optimization, significantly contributing to reduction of greenhouse gases from municipal waste and the resultant heat is convertible into electricity or useful heat. Furthermore, the use of high-tech incinerators can potentially reduce waste by 90% and make disposal of the residue or slag by incineration plants economically attractive as these materials may be used as substitute materials for grit and gravel for industrial uses.^13^ Landfilling is a ‘least preferred’ waste management technique as several nations have reached their landfill capacity resulting in the threat of an imminent waste management crisis occasioned by continued environmental degradation.^14^ Additionally, conventional landfilling is widely considered to be unsafe, as landfills harbor both harmful and non-biodegradable substances that could leach into water bodies. Consequently, the use of landfilling as a method of waste management has come under stringent scientific scrutiny leading to a radical reduction of the practice across Europe.^15^ Sustainable municipal waste management approaches such as recycling, mechanical-biological treatment and incineration are considered eco-friendly and economically efficient and effective for the conversion of waste into useful resources.^15^,^16^

**Abbreviations**
*GEMA*: Ga East Municipal Assembly
*GH¢*: Ghana Cedis

As a waste disposal method, composting promotes waste reduction, whiles its outputs are beneficial agricultural inputs in the form of manure and fertilizer.^17^,^18^ Biogas may be captured and further refined to obtain fuel (methane gas) during the composting process, which can be useful for domestic heating and cooking; however, the volume of biogas is limited by the biowaste composition.^19^ In spite of these ecologically sound waste management techniques, GEMA seems to be increasingly relying on landfilling as a waste management practice in the municipality. It is against this backdrop that this study was undertaken to characterize the generation of municipal solid wastes, identify waste management practices, and model waste flow in GEMA, determine recoverable resources and their market values based on sustainable waste-to-resource techniques, and recommend a more sustainable waste management approach for the municipality.

## Methods

### Study Area

**Figure 1:**
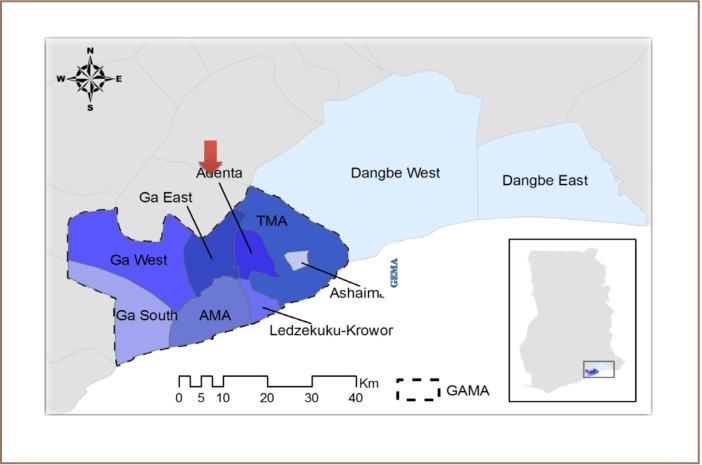
Map of Greater Accra Region and Ga East Municipal Assembly (GEMA)

The Ga East Municipal Assembly is one of 16 districts in the Greater Accra region of Ghana established in 2004 after the passing of the Legislative Instrument 2036. It was formally part of the Ga district assembly, located at the northern part of the Greater Accra Region with a total land area of 85 km^2^ (*Figure 1*). GEMA has an estimated population of 198,220 (51% males and 49% females) with an inter-censal growth rate of 4.2%, mainly as a result of migration inflows.^20^,^21^

Geographically, the municipality falls within the savannah agro–ecological zone and is an urban municipality with 82% of the populace residing in the urban and peri-urban areas and 18% inhabiting rural areas. The municipality has 17 health facilities, 14 of which are privately owned, and 234 private and 67 public schools.^21^ GEMA was one of the hardest hit areas in a recent cholera outbreak.^22^,^23^ Farming is the major economic activity for the population, with about 55% of the population employed in the agricultural sector, followed by the service, industrial and construction sectors. The problem of waste is even more acute due to an increasing population and the low socio-economic status of most inhabitants of the municipality.^24^ The poor sanitary condition of the community led to the inauguration of a seven-year strategic Town and Environmental Sanitation Development Plan for crafting strategy to improve sanitation conditions through gradual reduction of indiscriminate disposal and littering.^25^

Abloradgei is one of the fastest developing settlements in GEMA and has a dump site described by the municipal assembly as a crude, un-engineered final disposal site of great environmental concern.^25^ The dump site is located 500 m west of a major hospital in the municipality and is quite noticeable due to its unpleasant odor from about 150 m away. The situation is worsened by the transport and dumping of waste from various municipalities by Zoomlion Ghana Ltd, a private waste management company.

### Study Design

This study employed an exploratory-descriptive case study using multiple cross-case analyses. Secondary data was reviewed to show the positive impacts of modern waste management approaches in various developed countries. The secondary data included data on composition of MSWs, MSW technologies, methods of MSW disposals, statistics on recoverable resources from MSWs, energy and heat generated from MSWs, common uses of recovered resources from MSWs and cost of components of MSWs were sourced from baseline studies, working papers of the international solid waste management association (ISWMA), municipal strategic waste management frameworks (Germany, Japan, USA, Switzerland, France, Austria), best practices and standards of MSWM, technical reports on solid waste management and research articles. A detailed analysis was performed of recoverable resources from collected solid waste in GEMA in order to determine their economic value. A semi-structured key informant interview guide was used to gather first-hand information from both residents and operators of the GEMA dumpsite (six key informants) through a Focus Group Discussion (FGD). The six key informants included the head of the municipal sanitation unit, the manager of the Abloradgei dumpsite, and 4 members of the municipal waste management team. Questions were asked on current general composition of MSW, mode of collection and disposal, record on poor sanitation-related diseases, use of modern treatment plant, waste management legislation and enforcement challenges, number of trucks received by the dumpsite per day, record on pretreatment of MSW before disposal, use of personnel protective equipment by workers of the dumpsite. The semi-structured interview questions can be found in Supplemental Material 1.

### Data Analysis

Qualitative trend analysis of the interviews for each topic was used to identify the major issues for each of the main themes and sub-themes. Wastes were sorted and characterized at collection and final disposal sites (Abloradgei) by the research team with the assistance of volunteer workers from GEMA. Microsoft Excel was used to collate and compute study components. The e!Sankey Pro software program (version 3.2) was employed to determine the waste material flows of the municipality. These estimations were arrived at by taking into account the prices of the recyclable materials per ton in Ghana Cedis (GH¢) multiplied by the quantity or weight of the materials generated by the municipality. It was assumed that the organic components of the waste could be processed into biogas. Thus, the potential biogas yield for the organic component of the waste was estimated and the price of natural gas per 1000 ft^3^ was used to obtain the value of this gas.

### Ethical Statement

We sought permission from the director of the municipal health management team and the district assembly responsible for waste management in the municipality. The objective of the study was explained to all respondents, after which written and verbal consent was received from each participant. All participants were assured of anonymity and confidentiality of the information obtained from them.

## Results

### Waste Management Practices in GEMA

**Figure 2:**
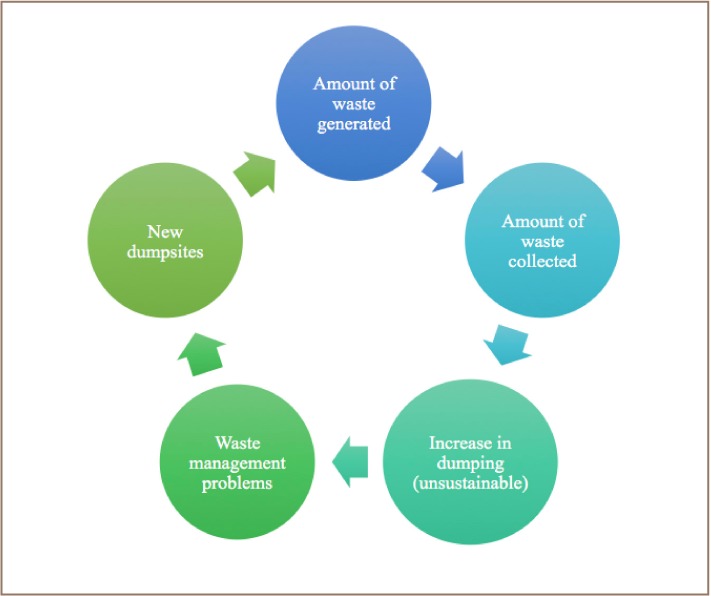
Waste management system in GEMA

**Figure 3:**
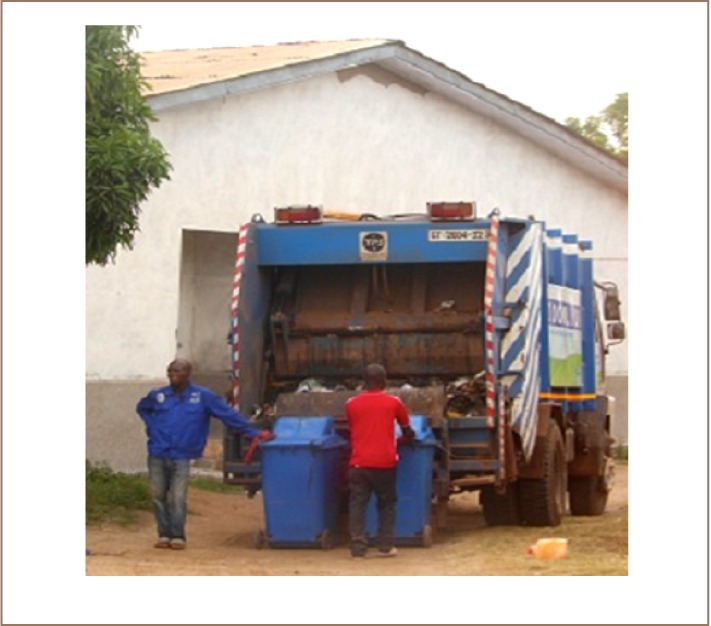
Door-to-door waste collectors emptying waste bins into waste trucks.

Solid waste management practices in GEMA are typical of those in most developing countries. With increasing population, waste generation in the municipality has increased and there is a lack of sustainable management practices. Wastes are collected by municipality staff and sent to the dump or landfill site. When a dumpsite becomes full, a new one is sited and the cycle continues as a causal loop cycle (*Figure 2*). Unsorted wastes are temporarily stored in these bins before their collection by the waste trucks (*Figure 3*).

**Figure 4:**
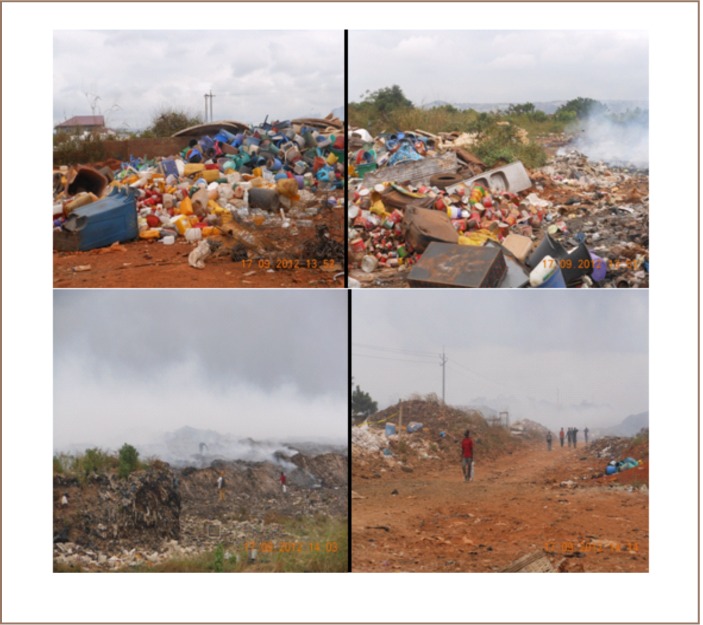
Snapshots from the Abloradgei dumpsite

Additionally, households use a range of other storage containers including polyethylene bags, metal buckets and broken plastic buckets. Some households also wrap waste materials in small polyethylene bags before placing the waste into bins. The net weight of dumped waste from the trucks ranges between 6.8 tons and 12.5 tons.^26^ On average, about 625 tons of waste are dumped at the site on a daily basis, amounting to 228,125 tons annually. Workers at the site disclosed that dumped waste at the site includes glass, polyethylene terephthalate (PET) bottles, plastic chairs and bowls, 5 liter plastic containers commonly called ‘gallons’, and metal scraps. There were over 20 waste scavengers (7 female, 13 male) working at the dumpsite (*Key Informant Interview and Focus Group Discussion*). Scavengers collect metal scraps from electronic wastes and sell them to middle men who transport them to factories in Tema (industrial city in Ghana) for melting and possible recycle or export.

### Solid Waste Management Flow in GEMA

**Figure 5:**
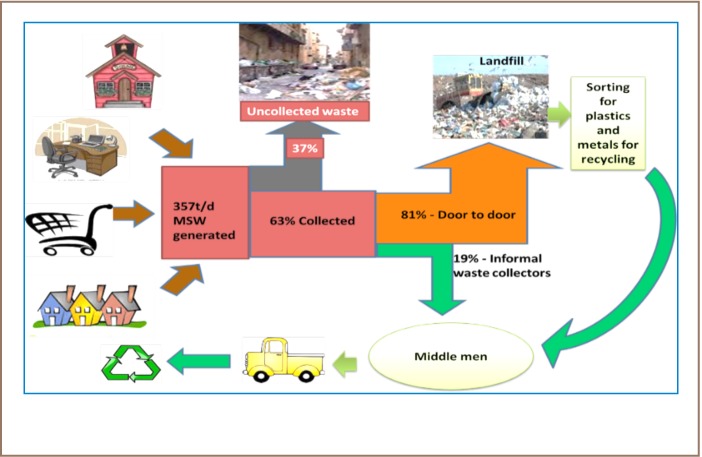
Solid waste management flow chart for GEMA Abbreviation: t/d, tons/day

**Figure 6:**
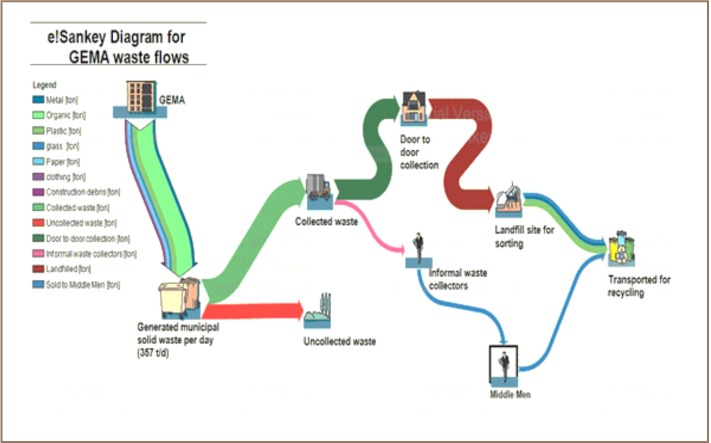
Generated e!Sankey model of waste _ows in GEMA

To understand the solid waste management system in the municipality, a material flowchart was generated using the e!Sankey software program (*Figures 5 and 6*). The waste flow in GEMA is not significantly different from that in other municipalities in developing countries. About 130,305 tons of municipal solid waste is generated yearly, mainly by households, markets, schools and offices in GEMA. Even though 82,092 tons (63%) of the waste are collected, a significant amount (48,213 tons per year) is left either on the streets or at unattended collection points. Consequently, they are later blown away by the wind; littering surroundings, piling up in gutters and open drains and causing floods during the rainy season.^7^

### Potential of Municipal Solid Waste in GEMA

**Figure 7:**
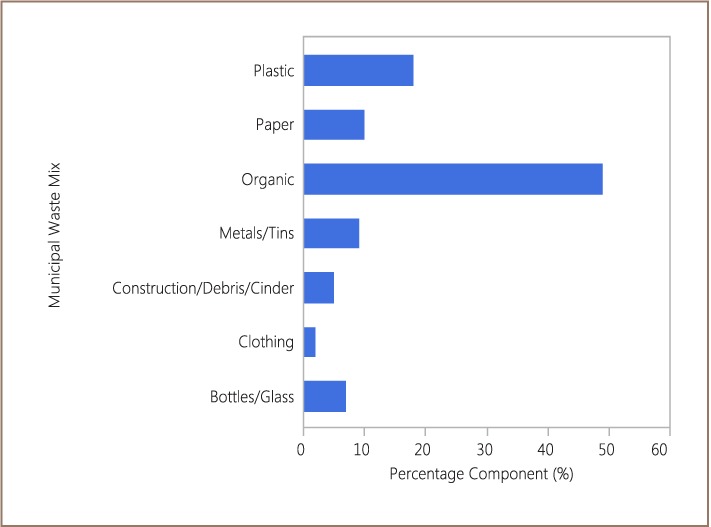
Municipal Solid Waste in GEMA with potential for resource recovery

Analysis of the municipal waste composition shows that many materials could be recovered for reuse.^7^ The municipal waste of GEMA contains at least seven type of materials (organic, glass, debris, clothing, metals, paper, and plastics) which could be recovered by means of various technologies (*Figure 7*). Organic waste constitutes the largest (49%) component of the total waste mix (*Figure 7*). About 44% of the waste mix is non-degradable and can be recycled or re-used for several purposes (*Figure 7*). The solid waste mix in GEMA includes 9% metals. These metals include aluminum cans, iron scraps and other metallic waste. Aluminum is produced from bauxite and used to produce cans and other materials for household consumption.

### Economic Value of Municipal Solid Wastes at GEMA

Table 1 shows the annual economic value of the waste composition in GEMA. Constituting 49% of the total municipal solid wastes of GEMA with a total weight of 63849.5 tons, the organic component has a biogas value of 2,106,339.2 m^3^ per annum. Thus, GH¢1,997,972.17 (US$503,909.40) is calculated to be the potential value of this resource.

**Table 1:** Economic Value of Recoverable Resources in MSW in GEMA Per Year Various prices per ton of waste materials were sourced from references 37–39

**Waste Material**	**%Total Waste**	**Weight (Tons)**	**Value/Ton (GH¢)**	**Total cost (GH¢)**
Plastic	18	23454.9	400^37^	9,381,960
Glass (mixed glass)	7	9121.4	108^38^	985,111
Paper	10	13030.5	396^39^	5,160,078
Metals	9	11727.5	988^37^	11,586,770

**Total**		57334.3		**27,113,919**

1 USD = 3.96 GH¢

To derive the value of clothing and debris components, it was assumed that the components would be fired in a combined heat and power plant to generate electricity and heat. The calorific values for the components were 14.6 MJ/kg for clothing and 8.4 MJ/kg for debris.^26^ The calculations assumed the following: 3.6 MJ = 1 kwh; and the combined heat and power plant had an efficiency of 45% for electricity generation and 37% for heat generation. This resulted in an annual potential electricity and heat of 11579 Mwh (1.32 Mw) and 9535 Mwh (1.09 Mw), respectively.

## Discussion

Waste pickers move from house to house collecting plastics, glass bottles, metal scraps and electronic waste. This door–to–door mode of collection accounts for 81% of the collected solid waste. In addition, containers are placed at vantage points in the communities and at market places. There is a private public partnership agreement between the Accra Metropolitan Assembly and Zoomlion Ghana Limited that register households for door-to-door collection with ‘standard’ (either 1.2 liters or 2.4 liters—depending on the anticipated amount of waste generated by the household) plastic waste bins provided free of charge. A monthly fee is paid for the collection of the waste.

It is worth noting that only 63% of the waste is collected and the rest is either left uncollected or dumped off indiscriminately in gutters and other open spaces or blown by the wind, littering the surroundings. Waste can be blown into and choke open drains, a leading cause of flooding in rainy seasons. Even though other municipalities have employed composting plants and recycling of plastics and metals, there is no record of recycling of the waste by the GEMA municipality. The final destination of the collected waste is the main waste dump site at Abloradgei. The Environmental Protection Agency of Ghana recently announced a momentary closure of the site as it is almost at capacity, and due to the concerns of neighboring occupants.^21^,^24^ The Abloradgei dump site (also known as Pantang landfill site), which covered an area of fifty acres when it first opened, has been reduced to about 10 acres due to the sale of some of the land to developers, as well as encroachment. Zoomlion Ghana Ltd. has managed the site for about six years and performs weekly spreading of piled-up refuse by bulldozers, occasional spray of the area with disinfectant, and facilitates fire prevention. In addition to the company's waste collectors, fully loaded waste collection trucks from other waste management companies and other organizations also empty their wastes at the site (Addo, 2015: *personal communication*). We recommend introduction of ‘spot fines’ for indiscriminate waste handling and disposal, along with encouraging households and businesses to compost organic waste for use on home gardens and fields, and selling polyethylene bags at shops as part of a polluter pay policy.^7^

Scavengers earn on average GH¢8 (US $4.2) daily, nearly twice the national minimum wage of GH¢4.48.7 However, because they do not wear any protective clothing at work, they are prone to numerous health problems, including frequent eye and body pains, headache, colds, cuts and burns due to their exposure to smoke from intentionally set fires and explosions on the site.^24^ It was also observed that the scavengers have very darkened skin, which can be ascribed to their continuous exposure to the smoke and dangerous gases on the site (*Figure 4*). In light of this, GEMA's waste management practices may be described as inconsistent with best practices where both households and manufacturers are responsible for waste collection and/or return to waste collection points.^16^,^28^ Households and businesses should be encouraged to sort their trash and remove compostable organic materials (metal, plastic, glass, and paper), and GEMA should provide protective clothing for waste collection staff and require its use while on duty, as well as provide more waste bins.

The e!sanky flowchart generated for waste flow affirms that GEMA's waste management practices are inconsistent with best practices of sustainable waste management.^16^,^29^ There is no recycling of waste generated in the municipality. Although some scavengers travel from house to house collecting metal scraps, these are not documented and are thereby overlooked. GEMA should incorporate informal waste collectors and scavengers into a new system, educate and encourage households and businesses to sort trash and remove compostable organic materials (metal, plastic, glass, and paper), and increase the number of waste collection vehicles and frequency of collection services.

GEMA still engages in conventional landfilling, which contributes to release of odors and gases, including methane gas, a major greenhouse gas, and a contributor to climate change and air pollution. Continuous dumping would require more space. GEMA could engage in pre-treatment before landfilling, as is performed in Germany.^28^ In addition, compost and biogas could be produced from organic wastes.^7^,^8^,^30^ Most households in GEMA depend upon charcoal, firewood, and fossil fuels (gasoline, diesel, and kerosene) for daily activities due to recurrent national power fluctuations. Composting and biogas production from these wastes could save agricultural land from pollution, enhance crop production, reduce reliance on fossil fuels, and decrease use of firewood, thus slowing deforestation. Demand for energy has become a grave concern to all Ghanaians, especially as economic activities continue to grow with and increasing population. The use of mechanical-biological waste treatment techniques to optimize feedstock for energy generation from waste could serve as a substitute for fossil fuel and diversify the monopoly of hydropower.^7^,^31^ GEMA should contract with businesses to accept and haul or ship sorted recyclable materials (metal, glass, paper, plastic, and electronics) to legitimate recycling companies.

As a result of a lack of waste disposal education and proper solid waste management system in GEMA, most wastes are not sustainably treated. One of the most important issues is that these wastes are not segregated at the source before reaching the dumpsite. This practice is not consistent with best practices where households and waste generators have the responsibility to segregate wastes.^28^,^32^ It is also at variance with the principle of “zero waste” which recommends that waste management processes be systematically designed to avoid and eliminate toxicity of waste materials to conserve and recover all resources. We recommend the use of mass media for education of the public on the harm caused by uncontrolled trash disposal.

Recovery of aluminum, iron and other metallic packaging would help reduce over-mining of aluminum and other metals and contribute to sustainability. Paper and glass recycling would greatly reduce dependence on raw materials. Recycling of plastic waste from GEMA would make a significant impact on the socio-economic development of the municipality since it would enhance conservation of natural resources and reduce greenhouse gases, especially the use of plastics to produce diesel fuel.^1^ Youth employment would also benefit from greater investment in waste management and the recycling industry. Companies would have access to local packaging materials and this would reduce importation of these materials from other parts of the world.

Even though global bauxite deposits are reported to be “the third most abundant metallic element found in the Earth's crust and could last for another 300 years”, aluminum recycling is very important to sustainable development and Ghana's economy.^33–35^ Deforestation, displacement of rural communities, human rights violations, soil degradation, release of toxic materials into the atmosphere, increased public health risk and extinction of organisms are among the adverse effects of the exploration and production of aluminum globally. Aluminum wastes are 100% recyclable and require only 5% of the total energy used to extract the alumina ore for processing into finished products.^33^ This suggests that GEMA and Ghana would profit significantly in terms of energy savings and the promotion of eco-friendly communities. Use of aluminum frames, doors and furniture would also reduce the reliance on timber for these purposes.^33^ The quality of PET bottles and other plastics decreases with every processing cycle and eventually “downcycling” would have to be practiced after a number of recycles. Experience in developed nations suggests that this type of recycling remains attractive to new investors when waste collection and sorting are standardized.^7^,^36^ In addition, creating aluminum and metal products from recycled wastes requires significantly less energy than mining.^37^ Furthermore, recycling can be repeated infinitely as the quality of such materials do not deteriorate from reprocessing.

Clothing waste made up the smallest component (2%) of the municipal solid waste in GEMA. However, clothing is deposited directly into the landfill at the dumpsite. When properly sorted by the community, discarded clothing could be used to benefit those in need, for example, orphanages. Other sorted grades may be used as insulation in furniture, cars and in the construction industry. These community recycling practices could reduce the amount of chemicals and water used for cotton farming and fabric processing. These practices would reduce the water burden and frequency of community water supply shortages. Glass and debris in the municipal solid waste in GEMA are presently ‘landfilled’ at the dumpsite. Production of glassware using recycled glass can save energy compared with their production from raw materials. Recycled glass, however, has a moderate market value and must be sorted into colors prior to melting. Debris and demolition waste can be crushed into gravel for reuse in road construction and landscaping.

In 2013, the annual average price of natural gas was $10.33 per thousand cubic feet according to the US Energy Information Administration, equivalent to GH¢26.86.^38^ Hence, 49% of the total municipal solid waste of GEMA, with a total weight of 63849.5 tons, could generate 74,384,667.5 ft^3^ (2,106,339.2 m^3^) of biogas with a value of GH¢1,997,972.17 ($768,393.62). A study conducted in Nigeria found that 39.1 million tons of organic municipal solid waste had the potential of generating 1.29 billion m^3^ of biogas, which corroborates the findings of the current study.^39^

The initial setup cost of a combined heat and power plant can be recovered from the first seven years of income generated by the plant. Considering that the typical life span of such plants is between 15–25 years with a minimal maintenance cost, the setup of these plants is economically attractive.^40^ This type of initiative would not only improve public health and the environment, but also provide additional income and more jobs.^7^ Nonetheless, it requires a commitment by GEMA's administration to craft the necessary policies, initiate practical implementation strategies and guidelines, and educate the citizenry.

## Conclusions

Effective and efficient waste management has been an important part of socio-political policies across the globe. In developed countries such as Germany, Japan, the United States, and European Union countries, waste is not simply discarded, but is considered to be a resource from which valuable materials can be derived. This has led to rigorous and stringent policies at the community/municipal, national and sub-regional levels, ensuring that the economic value of municipal waste is considered and processed to recover resources for reuse without compromising the needs and demands of future generations. On the contrary, GEMA's waste management system has underexploited the necessary mechanisms that could promote closed-loop waste management.

Waste collectors move from house to house, collecting only from households who signed up for this service. GEMA lacks the necessary modern waste management infrastructure and the only available method of treating waste is the conventional method of landfilling and surface dumping. The waste mix of GEMA shows that its recyclable components possess significant market value. If the recyclables would be separately collected and sold, a total annual revenue of GH¢27,113,919 ($10,845,568) is projected. The organic components could be used for biogas (2,106,339.2 m^3^) production with a market value of GH¢1,997,972.17 (US$768,393.62) per annum. The clothing and debris components could be incinerated for electricity and heat generation (1.32M w, 1.09M w yearly, respectively). Establishment of a resource center in the municipality is recommended to ensure a circular economy and improve sustainable waste management. We also recommend GEMA enact and enforce sanitation by-laws. A summary of our recommendations can be found in Supplemental Material 2.
