# Supplementary material for: Resource Prospects of Municipal Solid Wastes Generatedin the Ga East Municipal Assembly of Ghana
Source: J Health Pollut. 2017 Jun 22;7(14):37–47. doi: 10.5696/2156-9614-7.14.37 (PMC6259481; doi:10.5696/2156-9614-7.14.37)
Supplement: Supplementary file 1 [file Ahiakpa_SuppMaterial1.docx]

**Supplemental Material 1**

**Structured interview guide for Ga East Municipal Assembly (GEMA)**

Questions asked about the waste management system of GEMA:

*SECTION A: Questions to Waste Management Department in GEMA*

1. What is the current general composition of the municipal solid waste?
2. What are the modes of collecting waste for transfer to the dumpsite?
3. Has there been any record on poor sanitation-related diseases in the municipality?
4. Does the municipality employ a modern treatment plant to transform waste to valuable resources?
5. What is the current situation on waste management legislation?
6. Do you employ the policies in this legislation and what are the challenges in implementing them?

*SECTION B: Questions to dumpsite workers in GEMA*

1. How many trucks does the dumpsite receive per day?
2. How do you pre-treat municipal solid waste at the dumpsite before dumping?
3. Do other municipalities also use this dumpsite?
4. Does the municipality employ other methods of waste management techniques apart from dumping?
5. Do you use personal protective equipment when working at the dumpsite?
