# Supplementary material for: Resource Prospects of Municipal Solid Wastes Generatedin the Ga East Municipal Assembly of Ghana
Source: J Health Pollut. 2017 Jun 22;7(14):37–47. doi: 10.5696/2156-9614-7.14.37 (PMC6259481; doi:10.5696/2156-9614-7.14.37)
Supplement: Supplementary file 2 [file Ahiakpa_SuppMaterial2.docx]

Supplemental Material 2

Summary of proposed sustainable waste management recommendations for GEMA

| **Waste management system** | **Soft infrastructure** | **Hard infrastructure** |
| --- | --- | --- |
| Generation and Disposal | - Education - Introduction of spot fines for indiscriminate waste handling and disposal - Forming “green clubs” in schools and communities | - Waste sorting from source - Selling polythene bags at shops - Recycling polythene bags into waste sorting collection bags |
| Collection of Waste | - Employ informal waste pickers to collect waste door-to-door | - Providing waste bins - Providing enough protective clothing for collection staff |
| Transportation | - | - Increasing collection vehicles and frequency of collection |
| Treatment and Disposal | - Employ scavengers and train to work at waste resource center - Enactment and enforcement of by-laws | - Waste management resource center |
